# Hybrid Imitation Learning Framework for Robotic Manipulation Tasks

**DOI:** 10.3390/s21103409

**Published:** 2021-05-13

**Authors:** Eunjin Jung, Incheol Kim

**Affiliations:** Department of Computer Science, Kyonggi University, Suwon-si 16227, Korea; isk03276@gmail.com

**Keywords:** robotic object manipulation task, hybrid imitation learning, behavior cloning, trajectory cloning, dynamics modeling

## Abstract

This study proposes a novel hybrid imitation learning (HIL) framework in which behavior cloning (BC) and state cloning (SC) methods are combined in a mutually complementary manner to enhance the efficiency of robotic manipulation task learning. The proposed HIL framework efficiently combines BC and SC losses using an adaptive loss mixing method. It uses pretrained dynamics networks to enhance SC efficiency and performs stochastic state recovery to ensure stable learning of policy networks by transforming the learner’s task state into a demo state on the demo task trajectory during SC. The training efficiency and policy flexibility of the proposed HIL framework are demonstrated in a series of experiments conducted to perform major robotic manipulation tasks (pick-up, pick-and-place, and stack tasks). In the experiments, the HIL framework showed about a 2.6 times higher performance improvement than the pure BC and about a four times faster training time than the pure SC imitation learning method. In addition, the HIL framework also showed about a 1.6 times higher performance improvement and about a 2.2 times faster training time than the other hybrid learning method combining BC and reinforcement learning (BC + RL) in the experiments.

## 1. Introduction

An advanced service robot automates tasks performed by humans in the past—recognizing the surrounding conditions correctly, including human motion, the position of objects, and obstacles. As intelligent service robots are increasingly finding their foothold in our daily lives, techniques for efficient robotic object manipulation are attracting much research attention [1]. Several researchers have studied motion planning, which is the mainstream approach to executing robotic manipulation tasks [2,3,4]. However, motion planning has several limitations. It requires highly sophisticated kinematics and an inverse kinematics model in a high-dimensional workspace to generate a control sequence for a multi-joint robotic hand [5]. Therefore, in an actual execution environment with perceptual and behavioral uncertainties, motion planning is prone to task failure, requiring frequent replanning [6]. Recently, there has been a surge in research on robotic manipulation learning, where frameworks based on machine learning and artificial intelligence as alternatives to motion planning are used to address these limitations [7,8]. Robotic manipulation learning also faces hurdles to its full implementation. Above all, object manipulation tasks using a multi-joint robotic hand require physical control of each joint motor in a high-dimensional continuous state–action space. Therefore, robotic manipulation learning involves a long learning period and a large amount of experience data. Additionally, in a real-world robotic manipulation environment, sensory data and action control have a high level of uncertainty. This environmental uncertainty should be addressed by learning flexible and reliable action policies during learning.

**Figure 1:**
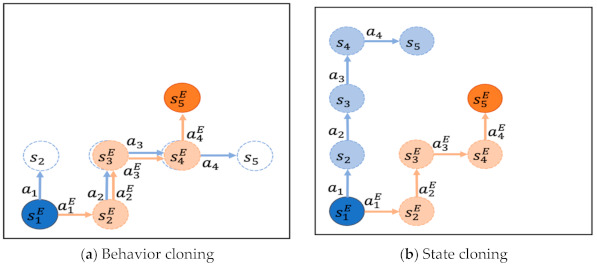
Behavior cloning (BC) vs. state cloning (SC).

Considering all these difficulties, roboticists are focusing their efforts on developing techniques to facilitate imitation learning of manipulation tasks [9,10]. Behavior cloning (BC) and state cloning (SC) are two typical imitation learning methods whereby robots learn action policies without any trials using a demo dataset containing human expert trajectories or other robotic task demonstrations. The policy network is taught to the learner robot by imitating the action sequences on demo task trajectories in BC [11,12,13] and following the task states similar to demo states on demo task trajectories [14,15,16]. The BC and SC training modalities are briefly illustrated in Figure 1a,b, respectively. The orange nodes and arrows represent the demo states $s_{t}^{E}$ and demo actions $a_{t}^{E}$ over a demo task trajectory.

In BC, as illustrated in Figure 1a, the learner robot starts from the demo state $s_{1}^{E}$ and updates the policy network, as it proceeds with the task, to reduce the difference between its intended action $a_{t}$ and the demo action $a_{t}^{E}$ assuming that a demo state $s_{t}^{E}$ is at each task step t, according to the demo task trajectory $d=\langle \left(s_{1}^{E},a_{1}^{E}\right),\;\left(s_{2}^{E},a_{2}^{E}\right),\ldots ,\;\left(s_{l}^{E},a_{l}^{E}\right)\rangle$. For example, let the action taken by the robot at the demo state $s_{1}^{E}$ be $a_{1}$ and the demo action be $a_{1}^{E}$. The policy network is then updated in BC to match the robot’s action$\;a_{t}$ to the demo action $a_{1}^{E}$. The robot does not proceed to the next step in the new task state $s_{2}$ but at the demo state $s_{2}^{E}$. As such, BC has the advantage of efficient learning of action policies, minimizing trial and error, but the disadvantage of learning action policies with limited coverage.

In SC, as illustrated in Figure 1b, the learner robot starts from state $s_{1}^{E}$ and proceeds with the task, updating the policy network to reduce the difference between the task state $s_{t+1}$, which the robot reaches by choosing and implementing its action$\;a_{t}$ at each task step t, and the demo state $s_{t+1}^{E}$, according to the same demo task trajectory d. For example, the policy network is updated in a manner that the task state $s_{2}$, which can be reached by the robot by taking action $a_{1}$ at the demo state $s_{1}^{E}$, matches the demo state $s_{2}^{E}$. Subsequently, instead of going on to the next step at the demo state $s_{2}^{E}$, the robot continues at a new task state $s_{2}$. As such, SC has the advantage of more flexible learning of action policies with broader coverage but the disadvantage of requiring more training time and computational effort. Moreover, stable training of the policy network is undermined if the learner robot’s real task trajectory $d^{\prime }$ is too far away from the demo task trajectory *d* while carrying out SC, as shown in Figure 1b.

There are recent works on hybrid learning methods combining BC imitation learning and reinforcement learning to improve both training efficiency and policy flexibility [17,18,19]. The hybrid learning methods make use of a combination of loss functions [17,18], a combination of reward functions [19], or pretraining and fine-tuning [20]. The first hybrid learning method [17,18], combining the BC loss $L_{BC}$ and the reinforcement learning loss $L_{RL}$, shows high efficiency of training, since it depends mainly upon the successful demo trajectories. However, the hybrid learning method with a loss combination reveals low flexibility of the learned policy, since extra experience data uncovered by the demo dataset cannot be used for training the policy network. The second hybrid learning method [19], combining the imitation reward $R_{BC}$ and the task reward $R_{RL}$, shows high flexibility of the learned policy, since it allows extra experience data to be used for training the policy. However, the hybrid learning method with a reward combination reveals low efficiency of training due to allowing trial-and-error experiences. The third hybrid learning method [20] pretrains the same policy network with BC and then fine-tunes it with reinforcement learning. It has the advantage that it can warm start the policy network, but it has a disadvantage that it might lead to poorer generalization [17]. To the best of the authors’ knowledge, no research has yet been devoted to developing a hybrid learning method that combines SC imitation learning with other learning methods.

This study proposes a hybrid imitation learning (HIL) framework, which is a novel imitation learning framework characterized by integrating BC and SC in a mutually complementary manner. The HIL framework efficiently combines BC and SC losses using the adaptive loss mixing weight. The HIL framework also uses a pretrained dynamics network to enhance the efficiency of SC. Finally, the framework performs stochastic state recovery, in which the task state is transformed into a demo state on a demo task trajectory while performing SC to ensure stable training of the policy network. This paper presents the results of a series of experiments testing the performance of the proposed HIL framework in executing pick-up, pick-and-place, and stack tasks and thus demonstrates its high training efficiency and policy flexibility.

The contributions of this paper can be summarized as follows:

- The proposed HIL framework combines BC and SC to enhance the efficiency of robotic manipulation learning by providing the synergistic effect of their respective advantages, i.e., high learning efficiency and high policy flexibility.
- The framework efficiently combines BC and SC losses using the adaptive loss mixing weight, which is automatically adjusted according to the degree of policy convergence.
- The framework efficiently implements SC learning using a pretrained dynamics network that predicts the next state from the result of the implemented action in association with the policy network within the HIL framework.
- The framework performs stochastic state recovery while performing SC to ensure a stable training of the policy network.
- This paper presents the process for and results of a series of object manipulation experiments using a 9-DOF (degree of freedom) Jaco robotic hand, demonstrating the high learning efficiency and policy flexibility of the proposed HIL framework.

The rest of the paper is organized as follows: Section 2 reviews the literature related to the topic of this study. Section 3 explains the design of the novel HIL framework in detail. Section 4 describes the implementation of the proposed HIL framework and the process and results of the experiments testing its performance, and Section 5 summarizes the main findings and concludes the study.

## 2. Related Work

Several studies have been devoted to robotic imitation learning designed to enhance the training efficiency of robotic manipulation tasks with a high-dimensional continuous state–action space [21,22]. Imitation learning methods can be categorized according to the information type of the demo data for policy network training. Among the information types contained in demo data, i.e., the demo task trajectory, BC trains policy networks based on demo actions [11,12,13]. The major advantage of BC is its facility of policy network convergence with a small number of learning iterations [17]. However, BC has limited coverage of learned policies and a bias risk of training data because it uses only demo data for policy training [12,17]. To compensate for these problems, previous studies attempted methods of using the additional experience data, collected by the learner robot from its experience, for policy training [23,24,25] or requesting experts for additional demo data throughout the learner robot’s online learning [12,22,26,27].

In SC, another imitation learning method, a policy network is trained using the demo states contained in a demo task trajectory [14,15,16]. The learner robot does not need to act as written on the demo task trajectory. Instead, SC requires the execution of a given task by passing through the task states as similar to the demo states as possible. This provides SC advantages over BC because it can reflect various states and actions outside the demo task trajectory during policy training. For this reason, compared to BC, SC provides the learner robot with policy learning with more flexibility and a broader application range [15]. In SC, the policy network parameters should be updated based on the SC loss calculated by adding the difference between each pair of demo states and task states. Some researchers attempted to predict a real task state using a dynamics network [14,15,16]. These studies proposed methods for defining the action to be taken by the learner robot using a policy network and predicting the next task state, which is the result of the action taken, using a dynamics network. These methods can help achieve a higher data efficiency than those for observing the next state after executing an action in a real environment. However, it is a great challenge to learn the optimal dynamics network in a robotic manipulation environment with a high-dimensional continuous state–action space, with the dynamics network performance posing the problem of interfering with policy network training [28,29]. In order to effectively train the dynamics network in a robotic manipulation environment, the proposed HIL learning framework applies a novel stochastic state recovery technique during state cloning.

Likewise, a range of hybrid learning methods combining BC and other methods have also been attempted [17,18,19,20]. This type of hybrid learning can be categorized into methods using a mixed loss function [17,18] or a mixed reward function [19]. Policy networks were trained using the mixed loss function combining the BC loss and reinforcement learning loss in studies on imitation learning [17,18], and the mixed reward function combining the task progress-based task reward and the BC-based imitation reward in a study on reinforcement learning [19]. These and other studies have proposed a variety of hybrid learning methods, but no research has yet been devoted to developing a hybrid learning method combining SC and other learning methods. Different from existing hybrid learning methods, however, our HIL hybrid imitation learning framework tries to combine BC and SC to learn an effective robotic manipulation policy for the first time. Therefore, the HIL framework can have high efficiency of training as well as high flexibility of policy.

## 3. Methodology

### 3.1. Problem Description

**Figure 2:**
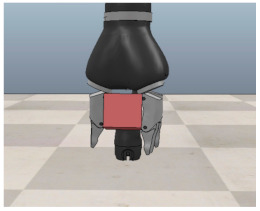
An example of robotic manipulation task.

In this study, the imitation learning problem for robotic manipulation tasks is defined as $M=\left(S,A,P,\;T,\;D,\;\pi \right)$. S and A denote the state space and action space, respectively, and both are defined as high-dimensional continuous spaces. For example, each state of the 9-DOF robotic hand executing a pick-up task, as shown in Figure 2, becomes a high-dimensional continuous space by expressing it in terms of 9-joint angles $\left(a_{j0},a_{j1},a_{j2},a_{j3},a_{j4},a_{j5},a_{j6},a_{j7},a_{j8}\right)$, angular velocities $\left(v_{j0},v_{j1},v_{j2},v_{j3},v_{j4},v_{j5},v_{j6},v_{j7},v_{j8}\right)$, and 7D pose of the manipulated object $\left(p_{x},p_{y},p_{z},o_{x},o_{y},o_{z},o_{w}\right)$. Moreover, the action space also becomes a high-dimensional continuous space by expressing the action executed by the robotic hand performing this task in terms of the joint velocity command ($v_{j0}^{cmd},v_{j1}^{cmd},v_{j2}^{cmd},v_{j3}^{cmd},v_{j4}^{cmd},v_{j5}^{cmd},v_{j6}^{cmd},v_{j7}^{cmd},v_{j8}^{cmd})$.

P denotes the stochastic state transition probability $P:S\times A\times S\to \left[0,\;1\right]$, which represents the effect of the action. It is assumed here that the stochastic state transition probability distribution is not known to the robot and that the robot must learn it by experience. T denotes the assigned robotic task, which is commonly expressed by pair $\left(c_{initial},c_{goal}\right)$ of the initial state condition ($c_{initial}$) and the goal state condition ($c_{goal}$). D denotes the demo dataset $D=\{\langle \left(s_{k_{1}}^{E},a_{k_{1}}^{E}\right),\ldots ,\;\left(s_{k_{l}}^{E},a_{k_{l}}^{E}\right)\rangle |k=1,\ldots ,n\}$ and consists of the sequence of the pairs$\;\left(s_{i}^{E},a_{i}^{E}\right)$ of the task trajectories performed by an expert.

In this study, it is assumed that the state and action spaces of the demo task performed by the expert are the same as those of the robotic learning task. From this, it follows that any given demo state and action $(s_{i}^{E}$ and $a_{i}^{E}$, respectively) contained in D are assumed to be $s_{i}^{E}\in S$ and $a_{i}^{E}\in A$. Conversely, π denotes the stochastic policy for the implementation of $T=\left(c_{initial},c_{goal}\right)$, expressed as $\pi_{T}:S\times A\to \left[0,\;1\right]$, which determines the action $a_{t}\;$to be taken by the robot in the state $s_{t}$ to execute the assigned task T. In this imitation learning problem M, the learner robot’s goal is to efficiently learn the action policy $\pi_{T}$ to achieve the task $T=\left(c_{initial},c_{goal}\right)$ using the demo dataset D. Table 1 summarizes the brief descriptions of variables used in this paper.

### 3.2. Hybrid Imitation Learning (HIL) Framework

**Figure 3:**
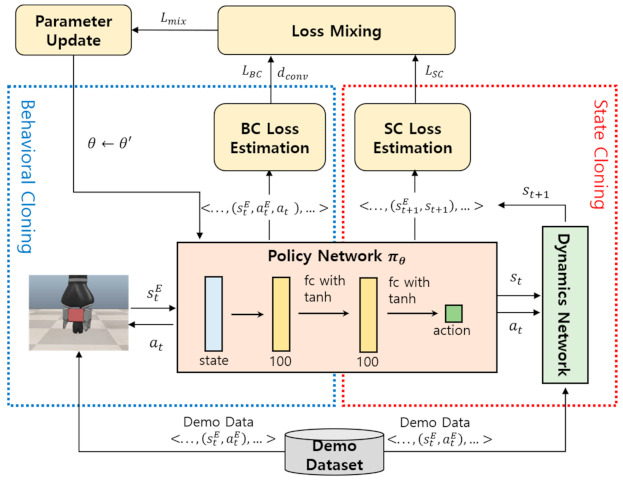
Hybrid imitation learning (HIL) framework.

BC and SC, two mainstream imitation learning methods, have shown excellent achievements in a variety of fields but have unique limitations. As explained previously, BC’s major problem is its failure to efficiently address various environmental states not contained in the demo dataset because its action policy learning is limited to the expert’s task trajectories contained in limited quantities in the demo dataset. By contrast, SC does not compel the learner robot to follow only the task trajectories contained in the demo dataset for action policy learning. Instead, SC allows the learner robot some free space to experience new states and actions not included in the demo dataset so that it can learn action policies with broader coverage and greater flexibility. The disadvantage of this approach is that it is time-consuming and cost-intensive because it allows the robot to learn through trial-and-error experience. In an attempt to overcome the drawbacks of these two imitation learning methods, this study proposes the HIL framework for efficient learning of robotic manipulation tasks. Figure 3 illustrates the overall architecture of the HIL framework.

The policy network, at the center of Figure 3, is a neural network module representing the action policy. In this study, a policy network $\pi_{\theta }$ was designed with three fully connected (fc) layers with tanh activation functions. Each layer contains 100 units. The BC part of the HIL framework includes a BC loss estimation module that estimates the BC loss$\;L_{BC}$ by following a demo task trajectory $\langle \left(s_{1}^{E},a_{1}^{E}\right),\;\left(s_{2}^{E},a_{2}^{E}\right),\ldots ,\;\left(s_{l}^{E},a_{l}^{E}\right)\rangle$ and comparing at each task state $s_{t}^{E}$ the action predicted by the policy network $a_{t}$ and the action taken using the demo data $a_{t}^{E}$. The SC part of the HIL framework consists of a dynamics network and an SC loss estimation module. The dynamics network is a neural network module that predicts the next environmental state $s_{t+1}$ after the learner robot has taken an action $a_{t}$ from any given state $s_{t}$. The SC loss estimation module starts from the initial state $s_{1}^{E}$ of the demo task trajectory $\langle \left(s_{1}^{E},a_{1}^{E}\right),\;\left(s_{2}^{E},a_{2}^{E}\right),\ldots ,\;\left(s_{l}^{E},a_{l}^{E}\right)\rangle$ and estimates SC loss $L_{SC}$ by comparing the next state predicted by the dynamics network $s_{t+1}$ and the next state of the demo task trajectory $s_{t+1}^{E}$ under the assumption that the policy network performs the predicted action $a_{t}.$ To efficiently integrate BC and SC, the HIL framework uses the mixed loss $L_{mix},\;$which combines the losses $L_{BC}$ and $L_{SC}.$ The loss mixing module (Figure 3, top) computes $L_{mix}$ by adaptively combining $L_{BC}$ and $L_{SC}$ at different ratios depending on the degree of policy convergence $d_{conv}.$ The degree of policy convergence $d_{conv}$ is a measure of whether the learner robot has learned the policy sufficiently to reproduce the demo task trajectory. Therefore, the adaptive loss mixing method of the HIL framework is designed to concentrate the learning on BC in the initial learning phase, where $d_{conv}$ is low, to teach it to reproduce the demo task trajectory. As $d_{conv}$ gradually increases, however, the learning focus is shifted to SC to include the ability to learn from experiences of various states and actions outside the demo task trajectory in policy learning. The overall learning process of the HIL framework is summarized in Algorithm 1.
**Algorithm 1.** Hybrid imitation learning framework.**Function HIL**(D, ρ)

/* the demo dataset $D=\{\langle \left(s_{k_{1}}^{E},a_{k_{1}}^{E}\right),\ldots ,\;\left(s_{k_{l}}^{E},a_{k_{l}}^{E}\right)\rangle |k=1,\ldots ,n\}$,

the state recovery probability ρ */

Initialize the policy network $\pi_{\theta }$ and the dynamics network $f_{\phi }$

$f_{\phi }$ = **Pretrain_Dynamics_Network**($f_{\phi },$ 
D, E)

**for** e = 0,…, E epochs **do**  **for** I = 0,…,|D| **do**
    Sample $d=\langle \left(s_{1}^{E},a_{1}^{E}\right),\;\left(s_{2}^{E},a_{2}^{E}\right),\ldots ,\;\left(s_{l}^{E},a_{l}^{E}\right)\rangle$ from D
    $L_{\mathrm{BC}},d_{\mathrm{conv}}=$ **Behavior_Cloning**($\pi_{\theta }$, d)

$L_{\mathrm{SC}}=$ **State_Cloning**($\pi_{\theta }$, $f_{\phi },\;d$, ρ)

$L_{\mathrm{mix}}=$ **Loss_Mixing**($L_{\mathrm{BC}}$, $L_{\mathrm{SC}},d_{conv}$)    Update the policy network parameters θ by gradient descent
         $\theta \leftarrow \theta -\nabla_{\theta }L_{\mathrm{mix}}$  **end for**

**end for**
**return**
$\pi_{\theta }$

After initializing the parameters of the policy network $\pi_{\theta }$ and the dynamics network $f_{\phi }$, the HIL framework pretrains the dynamics network $f_{\phi }\;$using the demo dataset D, followed by the iterative training of the learning process consisting of demo task trajectory sampling, BC, SC, loss mixing, and policy network updating. First, in the demo task trajectory sampling process step, a demo task trajectory $d=\langle \left(s_{1}^{E},a_{1}^{E}\right),\;\left(s_{2}^{E},a_{2}^{E}\right),\ldots ,\;\left(s_{l}^{E},a_{l}^{E}\right)\rangle$ is selected for training from D. In the BC step, $d_{conv}$ is computed based on the demo task trajectory $d=\langle \left(s_{1}^{E},a_{1}^{E}\right),\;\left(s_{2}^{E},a_{2}^{E}\right),\ldots ,\;\left(s_{l}^{E},a_{l}^{E}\right)\rangle$. In the SC step, $L_{SC}$ is computed based on d and $f_{\phi }$. In the loss mixing step, $L_{mix}$ is computed using the adaptive loss mixing weight $\alpha_{e}$, as

(1) $$L_{mix}=\left(1-\alpha_{e}\right)L_{BC}+\alpha_{e}L_{SC}$$

where the adaptive loss mixing weight $\alpha_{e}$ is not a constant but a variable equivalent to $d_{conv}$ ($\alpha_{e}=d_{conv})$; that is, $\alpha_{e}$ increases with increasing $d_{conv}$, resulting in a decrease in the weight of $L_{BC}$ and an increase in the weight of $L_{SC}$ in $L_{mix}$. Finally, in the policy network update step, the parameter θ of the policy network $\pi_{\theta }$ is updated based on the mixed loss, as expressed by Equation (2). In other words, in the HIL framework, θ of the policy network $\pi_{\theta }$ is updated using a gradient descent analysis of $L_{mix}$ with respect to the demo task trajectory.

(2) $$\theta \leftarrow \theta -\nabla_{\theta }L_{\mathrm{mixed}}$$

Algorithm 2 describes the implementation process of the aforementioned BC step of the HIL framework in detail. In BC, the learner robot follows the demo task trajectory $d=\langle \left(s_{1}^{E},a_{1}^{E}\right),\;\left(s_{2}^{E},a_{2}^{E}\right),\ldots ,\;\left(s_{l}^{E},a_{l}^{E}\right)\rangle$, concurrently selecting the action $a_{t}$ to be taken in each demo state $s_{t}^{E}$ using the policy network $\pi_{\theta }$ ($a_{t}\sim \pi_{\theta }\left(s_{k}^{E}\right))$ and computing the probability of $a_{t}$ to be selected in the state $s_{t}^{E}$ $\left(p_{t}=Ρ\left(a_{t}|s_{t}^{E};\pi_{\theta }\right)\right)$, followed by the calculation of $L_{BC}$ based on the $a_{t}$ determined by the policy network $\pi_{\theta }$ and the demo action $a_{t}^{E}$, as

(3) $$L_{\mathrm{BC}}=\sum_{\left(s_{t}^{E},{\;a}_{t},{\;a}_{t}^{E}\right)\in B}\|a_{t}-a_{t}^{E}\|{}_{2}^{2}.$$

Accordingly, $L_{BC}$ is defined as the sum of the differences between the chosen $a_{t}$, determined by the policy network $\pi_{\theta }$ in each demo state $s_{t}^{E}$, and the demo action $a_{t}^{E}$, based on the demo task trajectory $d=<\left(s_{1}^{E},a_{1}^{E}\right),\;\left(s_{2}^{E},a_{2}^{E}\right),\ldots ,\;\left(s_{l}^{E},a_{l}^{E}\right)$>. Additionally, the $d_{conv}$ of the policy network $\pi_{\theta }$ is calculated in the BC function based on the probability $p_{t}$ for the learner robot to select the action $a_{t}$ at each state $s_{t}^{E}$, as

(4) $$d_{conv}=\frac{1}{l}\sum_{t=1}^{l}p_{t}.$$

Lastly, $L_{BC}$ and $d_{conv}$ calculated as above are returned as the final result of the BC function.
**Algorithm 2.** Behavior cloning.**Function Behavior_Cloning**($\pi_{\theta }$, d)

/* the policy network $\pi_{\theta }$

the demo data $d=\langle \left(s_{1}^{E},a_{1}^{E}\right),\;\left(s_{2}^{E},a_{2}^{E}\right),\ldots ,\;\left(s_{l}^{E},a_{l}^{E}\right)\rangle$ */ Initialize the episodic buffer B to be empty

**for** t = 1,…, l timesteps **do**

Sample an action $a_{t}\sim {\;\pi }_{\theta }\left(s_{t}^{E}\right)$
            $p_{t}=Ρ(a_{t}|s_{t}^{E};\pi_{\theta })$
          $B\leftarrow B\cup \left\{\left(s_{t}^{E},{\;a}_{t}^{E},a_{t},p_{t}\right)\right\}$ **end for**
Estimate the BC loss $L_{\mathrm{BC}}$
          $L_{\mathrm{BC}}=\underset{\left(s_{t}^{E},{\;a}_{t},{\;a}_{t}^{E}\right)\in B}{\sum }\|a_{t}-a_{t}^{E}\|{}_{2}^{2}$

Calculate the degree of cloning $d_{conv}$          $d_{\mathrm{conv}}=\frac{1}{l}\overset{l}{\underset{t=1}{\sum }}p_{t}$

**return** $L_{\mathrm{BC}},d_{\mathrm{conv}}$

### 3.3. State Cloning with Dynamics Network

As explained above, in the HIL framework, both BC and SC are used to train the policy network $\pi_{\theta }$. Unlike BC, SC does not compel the learner robot to imitate the demo task trajectories only but provides opportunities to attempt new states and actions. In general, a learner robot faces difficulty in reproducing the demo task trajectories in a real task environment with a very wide state and action space and high environmental uncertainties. It is also at a high risk of facing new states unencountered during demo task execution, even if the same task is repeatedly performed. Therefore, in a robotic manipulation task environment, a learner robot must be allowed to experience new states and actions outside the demo task trajectories and to carry out SC imitation learning based on those experiences to learn more flexible action policies with broader coverage.

**Figure 4:**
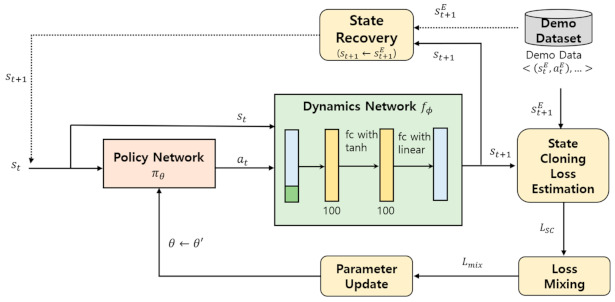
State cloning with dynamics network.

Simply put, SC is the process of updating the parameter θ of the policy network $\pi_{\theta }$ to reduce the loss between state $s_{t}^{E}$ and the learner robot’s task state $s_{t}$ on the demo task trajectory $d=\langle \left(s_{1}^{E},a_{1}^{E}\right),\;\left(s_{2}^{E},a_{2}^{E}\right),\ldots ,\;\left(s_{l}^{E},a_{l}^{E}\right)\rangle$. The SC process of the HIL process is illustrated in Figure 4. The figure shows that the SC in the HIL framework uses policy, dynamics, state recovery, SC loss estimation, loss mixing, and parameter update modules.

Suppose that a learner robot is currently performing the action$\;a_{t}$ in the environmental state $s_{t}$. The dynamics network $f_{\phi }$ consisting of three fully connected layers among the SC modules then predicts the next state $s_{t+1}$ as a consequence of the action$\;a_{t}$. Accordingly, if the policy network $\pi_{\theta }$ decides on an action $a_{t}$, based on the input of the current state $s_{t}$, the dynamics network $f_{\phi }$ predicts the next state $s_{t+1}$ based on the current state $s_{t}$ and action $a_{t}$. The dynamics network is used in the SC step of the HIL framework following pretraining using the demo dataset D. Pretraining the dynamics network greatly contributes to enhancing the learning efficiency of the policy network during the SC process.

The state recovery module plays the role of moving any given state task state $s_{t}$, which is not on the demo task trajectory $d=\langle \left(s_{1}^{E},a_{1}^{E}\right),\;\left(s_{2}^{E},a_{2}^{E}\right),\ldots ,\;\left(s_{l}^{E},a_{l}^{E}\right)\rangle ,$ to a state $s_{t}^{E}$ on the demo task trajectory during the learner robot’s task execution. In general, throughout the SC imitation learning, the learner robot is not instructed to imitate the actions on the demo task trajectory. This makes the learner robot’s actual task trajectory $d^{\prime }$ prone to digression from the demo task trajectory d. An excessive discrepancy between the demo task trajectory d and the actual task trajectory $d^{\prime }$ undermines stable learning of the policy network $\pi_{\theta }$. Therefore, in the state recovery module of the HIL framework, stochastic state recovery is intermittently performed to return the learner robot’s state to a state on the demo task trajectory using the recovery probability ρ.

The SC loss estimation module calculates $L_{SC}$ using the difference between the two states $s_{t}^{E}$ and $s_{t}$. The resultant $L_{SC}$ value is integrated into the mixed loss with $L_{BC}$ using the loss mixing module. To minimize the mixed loss, the parameters of θ of the policy network $\pi_{\theta }$ are updated using the parameter update module.

Algorithm 3 describes the SC learning process in detail. The SC learning process involves the demo task trajectory $d=\langle \left(s_{1}^{E},a_{1}^{E}\right),\;\left(s_{2}^{E},a_{2}^{E}\right),\ldots ,\;\left(s_{l}^{E},a_{l}^{E}\right)\rangle$, policy network $\pi_{\theta }$, state recovery probability ρ, and pretrained dynamics network $f_{\phi }$. It begins with task initialization in which the episodic buffer B is emptied, and the learner robot’s task state $s_{0}$ is set to the initial state $s_{1}^{E}$ on the demo task trajectory. This is followed by action sampling, the second step of SC in which the $a_{t}$ to be executed in the state $s_{t}$ is decided using $\pi_{\theta }$ ($a_{t}\sim \pi_{\theta }\left(s_{t}\right)$). The third step of SC is the next state determination, in which $a_{t}$ is executed, and the next task state $s_{t+1}$ is determined. Depending on ρ, the next state $s_{t+1}$ is either the state predicted by $f_{\phi }\left(s_{t},a_{t}\right)$ or a state on the demo task trajectory $s_{t+1}^{E}$. In the fourth step, the pair consisting of the determined next state $s_{t+1}$ and the demo state $s_{t+1}^{E}$ ($s_{t+1},s_{t+1}^{E})$ is stored in the episodic buffer B. Steps 2–4 are performed iteratively until task termination, followed by SC loss estimation, which is the final step. In the SC loss estimation step, $L_{SC}$ is calculated using the difference between the two states $s_{t}^{E}$ and $s_{t}\;$based on the data stored in buffer B, as

(5) $$L_{SC}=\underset{\left(s_{t},{\;s}_{t}^{E}\right)\in B}{\sum }\|s_{t}-s_{t}^{E}\|{}_{2}^{2}\;$$

**Algorithm 3.** State cloning.**Function State_Cloning**($\pi_{\theta }$, $f_{\phi },\;d$, ρ)

/* the policy network $\pi_{\theta },$ the pretrained dynamics network $f_{\phi }$,

the demo data d, the state recovery probability ρ */ Initialize the episodic buffer B to be empty
               $s_{0}\leftarrow s_{0}^{E}$

**for** t = 1,…, l−1 timesteps **do**

Sample an action $a_{t}\sim {\;\pi }_{\theta }\left(s_{t}\right)$
             $s_{t+1}\leftarrow \left\{\begin{array}{c} f_{\phi }\left(s_{t},a_{t}\right),\;\;\mathrm{with}\;\;\left(1-\rho \right)\\ s_{t+1}^{E},\;\;\;\mathrm{with}\;\;\rho \end{array}\;\right.$
B ← B ∪ {($s_{t+1},s_{t+1}^{E})\}$ //append each pair of states to B **end for**
Estimate the SC loss $L_{\mathrm{SC}}$
             $L_{\mathrm{SC}}=\underset{\left(s_{t},{\;s}_{t}^{E}\right)\in B}{\sum }\|s_{t}-s_{t}^{E}\|{}_{2}^{2}$

**return** $L_{\mathrm{SC}}$

### 3.4. Pretraining the Dynamics Network

As mentioned above, the dynamics network $f_{\phi }$ performs pretraining using the demo dataset D prior to implementation of SC in the HIL framework. Algorithm 4 describes the pretraining of the dynamics network.
**Algorithm 4.** Pretraining the dynamics network.**Function Pretrain_Dynamics_Network** ($f_{\phi },$ D)

/* the dynamics network $f_{\phi }$, the demo dataset D */

**for** e = 0,…,E epochs **do**
  **for** i = 0,…,|D| **do**
Initialize the episodic buffer B to be empty Sample $d=\langle \left(s_{1}^{E},a_{1}^{E}\right),\left(s_{2}^{E},a_{2}^{E}\right),\ldots ,\left(s_{il}^{E},a_{il}^{E}\right)\rangle$ from D

Initialize the environment to initial state $s_{1}^{E}$

**for** t = 1,…. il−1 timesteps **do**
Execute an action $a_{t}^{E}$ and perceive the next state $s_{t+1}$

B ← B ∪ {$\left(s_{t},a_{t}^{E},s_{t+1}\right)\}$//append each state transition to B    **end for**
Update the dynamics network parameters ϕ by gradient descent
      
$\phi \leftarrow \phi -\nabla_{\phi }\left(\underset{\left(s_{t},a_{t}^{E},s_{t+1}\right)\in B}{\sum }\|f_{\phi }\left(s_{t},a_{t}^{E}\right)-s_{t+1}\|{}_{2}^{2}\right)$   **end for**
**end for**
**return** $f_{\phi }$

Dynamics network pretraining begins with task initialization, emptying the episodic buffer B, sampling a demo task trajectory $d=\langle \left(s_{1}^{E},a_{1}^{E}\right),\left(s_{2}^{E},a_{2}^{E}\right),\ldots ,\left(s_{il}^{E},a_{il}^{E}\right)\rangle$ from the demo dataset D, and initializing the environmental state to the initial state $s_{1}^{E}$ of the demo task trajectory. In the second step, “action execution”, the demo action $a_{t}^{E}$ is executed in the current state $s_{t}^{E}$. In the third step, “state observation”, the next state$\;s_{t+1}$ resulting from the second step, i.e., execution of the demo action $a_{t}^{E}$, is observed, and these state transition experience data $\left(s_{t},a_{t}^{E},s_{t+1}\right)$ are stored in the episodic buffer B. The second and third steps are iteratively performed through the termination of the task, and the training data for the dynamics network are collected in the episodic buffer B. In the last step, “parameter update”, the parameters ϕ of the dynamics network $f_{\phi }$ are updated using the parameters of the training data $\langle \ldots \left(s_{t},a_{t}^{E},s_{t+1}\right),\ldots \rangle \;$ stored in the episodic buffer B, as

(6) $$\phi \leftarrow \phi -\nabla_{\phi }\left(\sum_{\left(s_{t},a_{t}^{E},s_{t+1}\right)\in B}\|f_{\phi }\left(s_{t},a_{t}^{E}\right)-s_{t+1}\|{}_{2}^{2}\right).$$

where $f_{\phi }\left(s_{t},a_{t}\right)$ and $s_{t+1}$ represent the next state predicted by the dynamics network $f_{\phi }$ and that observed in the real-life setting, respectively. Consequently, the parameter ϕ of the dynamics network is updated using a gradient descent optimization method based on a loss function defined as the difference between the next state predicted by the dynamics network $f_{\phi }\left(s_{t},a_{t}^{E}\right)$ and the next state actually observed $s_{t+1}$ in the parameter update step.

## 4. Implementation and Evaluation

### 4.1. Manipulation Tasks

**Figure 5:**
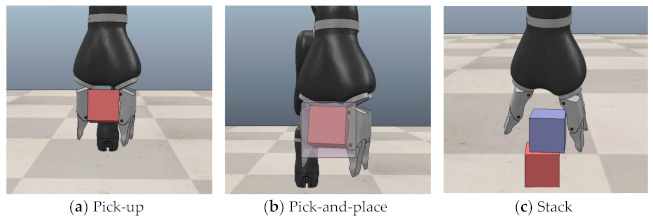
Three robotic manipulation tasks.

In this study, the performance of the HIL framework was analyzed by testing it on three types of robotic manipulation tasks (pick-up, pick-and-place, and stack tasks) using a 9-DOF Jaco robotic hand, as shown in Figure 5.

All blocks used for the three object manipulation tasks were assumed to be rigid cubes (5 cm^3^). Each task state is expressed in terms of the angle and angular velocity of the Jaco robotic hand joints and the position and orientation of the target block. Each control action of the Jaco robotic hand is expressed as a joint velocity command, which is a nine-dimensional integer vector. The uncertainty of the real world was simulated by applying Gaussian noise $N\left(0,\;0.001\right)$ to all actions of the Jaco robotic hand executed in the CoppeliaSim simulators. The characteristics of each of the three object manipulation tasks are subsequently summarized.

The goal of the pick-up task is to grab the target block placed on the table and lift it to or above the predetermined reference height of 20 cm. For this task, the initial location of the object block on the table was set at random, while the initial position of the robotic hand was fixed. This task has the smallest state space, and its task state is hence easy to predict by the dynamics network compared with the other manipulation tasks.

The goal of the pick-and-place is to grab the target block placed on the table and move it from the initial location to the predetermined target position. If the distance between the initial position of the target block and the target position is less than 5 cm, the task is considered successful. The initial and final locations of the target block were set at random for each task, with the initial position of the robotic hand again fixed to the same position. Compared to the pick-up operation, which requires simply lifting the object, this task is more challenging because it has a much larger state space and a longer task trajectory. It is less challenging than the stack task, which requires moving the target block and stacking it onto another block, although these two tasks are similar in that the target block must be moved to the target position.

The goal of the stack task is to grab the target block and stably place it on another block. The initial position of the target block is set at random for this task, while the initial positions of the robotic hand and the second block onto which the target block should be stacked are always fixed. Of the three tasks, this has the largest state space and highest complexity.

### 4.2. Model Training

The HIL framework was implemented in Ubuntu 18.04 LTS computing environments using PyTorch of the Python deep learning library. The policy network and dynamics network of the HIL framework were designed with three fully connected layers. Each layer consisted of 100 hidden units, and a rectified linear unit was used as the activation function. The robot manipulation task environments, including the Jaco robotic hand, were modeled using the robot simulator CoppeliaSim, and PyRep, a toolkit for robot learning.

The demo data for training were gathered by an iterative run of the sequential processes of motion planning, plan execution, and trajectory recording. In motion planning, all trajectories of the Jaco robotic hand to reach the target pose from the initial pose of each task were calculated using the motion planner Open Motion Planning Library plugin for the robot simulator CoppeliaSim. In the processes of planning execution and trajectory recording, the state–action pairs at each simulation timestep were recorded while the tasks were sequentially executed by the joints of the Jaco robotic hand according to the planned trajectories. As explained above, each state is expressed in terms of the position of the manipulation task objects and those of the robotic hand joints, and each action in terms of a joint velocity command for each joint of the robotic hand. Through this method, the recorded sequence of the state–action pairs throughout the demo task became the demo data representing the task episode, and a demo dataset was constructed by collecting them. The demo dataset for HIL consisted of 200, 250, and 300 task execution trajectories for pick-up, pick-and-place, and stack tasks, respectively, considering their increasing complexity. The demo dataset was divided into training and validation datasets at a ratio of 10:1, which were used for training and testing of the HIL framework.

The demo dataset was used to pretrain the dynamics network prior to its inclusion in the HIL framework, and the pretrained dynamics network was fine-tuned again in an end-to-end training process of the entire HIL framework. The policy network and dynamics network in the HIL framework were trained at a learning rate of $10^{-3}$ using the optimizer Adam [30].

### 4.3. Experiments in Simulated Envirionment

The experiments testing the performance of the HIL framework were conducted in a computing environment consisting of an Intel i9-7920X Processor 12-core 24-thread 2.9 GHz CPU and GeForce GTX 2080 Ti GPU. The first experiment aimed to prove the superiority of the proposed HIL framework through a performance comparison of various loss mixing methods combining $L_{BC}$ and $L_{SC}$. To this end, the mean task success rate and learning time (min) were compared while varying the value of $\alpha_{i}$, the mixed weight coefficient used in calculating $L_{mix}$ and $L_{SC}$ (see Equation (7)). A comparison was also made between the application cases of the fixed mixing weights (0, 0.3, 0.6, and 1.0) and the automatically determined adaptive loss mixing weight depending on the degree of BC $d_{cloning}$ ($\alpha_{i}=d_{cloning}$), as is the case with the HIL framework. In particular, mixing weights of $\alpha_{i}=0$ and $\alpha_{i}=1$ indicate the exclusive application of pure BC imitation learning and pure SC imitation learning, respectively. The state recovery probability ρ for the SC function was set to be 0.6.

(7) $$L_{mix}=\left(1-\alpha_{i}\right)L_{BC}+\alpha_{i}L_{SC},$$

**Figure 6:**
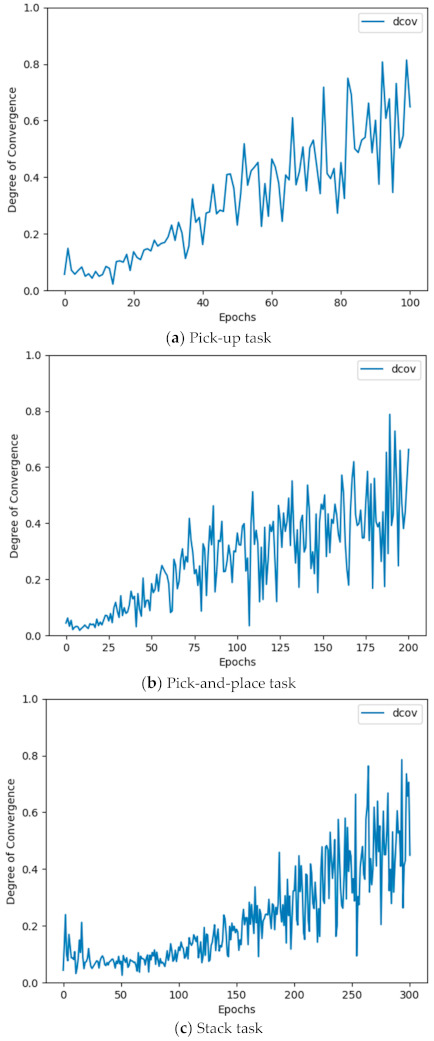
Changes in $d_{conv}$ for learning manipulation tasks.

Table 2 outlines the results of the experiment. The case using the adaptive loss mixing weight ($\alpha_{i}=d_{conv})$ outperformed the cases using a fixed mixing weight in all three manipulation tasks. Among the different mixing weight values, the case using SC loss only ($\alpha_{i}=1.0)$ outperformed the case using BC loss only ($\alpha_{i}=0)$ for action policy learning in all three manipulation tasks, presumably because SC allows the learner robot more opportunities than BC to experience the actions not included in the demo data for the learner robot to learn action policies with wider coverage and higher reliability than BC. Comparing the other two cases of fixed mixing weights ($\alpha_{i}=0.3,\;0.6)$, different performance levels were observed depending on the executed task type. In the pick-up task, the case with a comparatively low mixing weight ($\alpha_{i}=0.3$) outperformed the other case ($\alpha_{i}=0.6$). In the pick-and-place and stack tasks, the case with a comparatively high mixing weight ($\alpha_{i}=0.6$) outperformed the other case ($\alpha_{i}=0.3$). This suggests that high performance can be expected by setting a low mixing weight (=higher $L_{BC}$ contribution) for a task with low complexity, such as the pick-up task, and a low mixing weight (=higher $L_{SC}$ contribution) for a task with high complexity such the pick-and-place and stack tasks. As such, expecting a uniformly high performance is difficult when using a fixed mixing weight $\alpha_{i}$ for different types of tasks with different complexities. By contrast, the HIL framework, using the adaptive loss mixing weight while learning depending on the degree of policy convergence $d_{conv}\;\left(\alpha_{i}=d_{conv}\right)$, showed a uniformly high performance in various tasks with different complexities. Figure 6 shows how $d_{conv}\;$changes during task learning. For all three tasks, we find that $d_{conv}\;$increases as training proceeds. These experimental results prove the superiority of the HIL framework, achieved by implementing an adaptive loss mixing weight method.

The second experiment was conducted to observe the effect of the stochastic state recovery performed by the SC function in the HIL framework and analyze the performance changes depending on the value of the state recovery probability ρ. To this end, the average success rate from 10 runs was measured, with the values of the state recovery probability ρ set to 1.0, 0.6, 0.3, and 0. Table 3 outlines the results of this experiment.

Table 3 shows that all three tasks failed when stochastic state recovery was not applied (ρ = 0). Conversely, the task success rate exceeded 0.5 when stochastic state recovery was applied (ρ = 1.0, 0.6, 0.3), thus verifying the positive effect on learning of stochastic state recovery. HIL performed best at ρ = 0.3 in the pick-up task, where the state space is relatively narrow and the length of the task episode is short, and at ρ = 0.6 in the pick-and-place and stack tasks (wide state space and long task episode length). In general, when ρ is set high, whenever a digression from the demo task trajectory occurs during the SC learning process, it is returned to one of the states of the corresponding demo state trajectory. If ρ is set low, digression from the demo task trajectory is tolerated for a long time, resulting in more opportunities to experience new states and actions unencountered while performing the demo task. Therefore, to obtain high HIL performance, it is advantageous to set the state return probability ρ low in a pick-up task, which has a relatively low complexity, and high in a pick-and-place or stack task, which has a higher complexity. Conversely, setting ρ to the maximum value of 1.0 resulted in the lowest performance across all three tasks, presumably because the restraint on the states was not allowing for any digression from the demo task trajectory at every timestep during the learning process, resulting in the HIL framework learning the same things as in a pure BC process, neutralizing the advantages of the former over the latter.

The third experiment was conducted to evaluate the superiority of the proposed HIL framework by comparing it with typical robot manipulation task learning methods. Specifically, the performance levels of pure BC imitation learning, reinforcement learning (RL) using the PPO algorithm [31], hybrid learning with a mixed loss function combining the BC loss and the reinforcement learning loss (BC + RL), and the proposed HIL framework were compared. The state recovery probability ρ of the HIL framework was set to 0.3 and 0.6 for the pick-up, and pick-and-place and stack tasks, respectively. The mean task success rate of each learning epoch of the policy network was used as the threshold for success. The reward function for RL was designed as follows:

**Pick-up:**  A reward of +0.01 is awarded when the robotic hand approaches the object, +0.1 when it grasps it, and +1 when it lifts it to or above a certain height.
**Pick-and-Place:**  A reward of +0.01 is awarded when the robotic hand approaches the object, +0.1 when it grasps it, and +10 when it lifts and moves it to or above a certain height.
**Stack:**  A reward of +0.01 is awarded when the robotic hand approaches the object, +0.1 when it grasps it, +1 when it lifts it to or above a certain height, and +10 when it stacks it onto another object.

**Figure 7:**
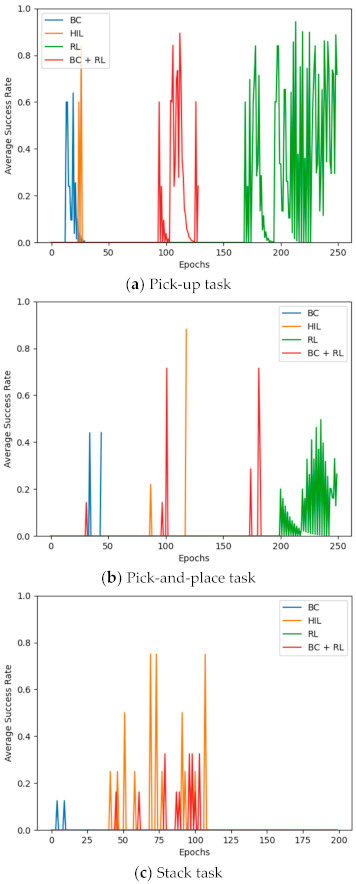
Comparison with conventional learning methods.

The results of this experiment are shown in Figure 7. This shows that pure BC improved the performance level most rapidly but was outperformed by the other two methods. This is because BC cannot reflect new states and actions not included in the demo data in the action policy and hence yields the lowest performance level in the manipulation tasks with high complexity and environmental uncertainty. On the other hand, RL using the PPO algorithm improved the performance level most slowly. In the stack task shown in Figure 7c, which had the highest complexity, RL provided no noticeable improvement for the first 400 epochs. For this reason, it is hard to distinguish the plot showing the RL result from the epoch axis in Figure 7c. From these experimental results, it can be assumed that RL requires a much higher number of trials than BC and HIL to learn a policy network to any significant extent, lowering the data efficiency. For all three tasks, the hybrid learning of BC + RL provided a faster improvement in the performance than pure RL. However, for two complex tasks such as pick-and-place and stack tasks, the hybrid learning of BC + RL showed a lower performance than the proposed HIL framework. By contrast, HIL showed the highest task performance improvement in all three manipulation tasks, presumably by fully benefiting from the combined advantages of BC with high learning efficiency and SC with various state–action experiences reflected in learning. The results of this experiment verify the high learning efficiency and task performance success rate of robots trained via HIL.

**Figure 8:**
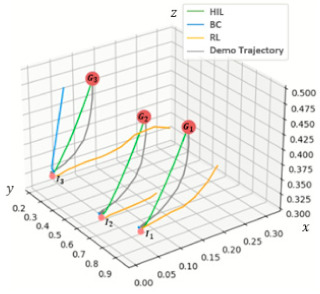
Qualitative comparison among the learned policies.

Finally, a fourth experiment was conducted to qualitatively evaluate the action policies resulting from HIL training. After 5 h of training time, the policies learned by BC, RL, and HIL were compared for the pick-up task. The object position changes observed when applying each policy to the real task are plotted in 3D space in Figure 8.

Figure 8 illustrates the trajectory of the target objects during task execution from their respective initial positions $I_{1},I_{2},I_{3}$ to their final poses $G_{1},G_{2},G_{3}$ for each of the policies learned. The object trajectories resulting from the policy learned through HIL, ($I_{1},\;G_{1}$), ($I_{2},\;G_{2}$), and ($I_{3},\;G_{3}$) in the three pick-up tasks, show that every action causing digression from the demo task trajectory during task execution is corrected to the demo task trajectory, allowing the robot to reach the target position. By contrast, the object trajectories resulting from the policy learned through BC show that digression from the demo task trajectory during task execution cannot be remediated. For example, in the ($I_{1},\;G_{1}$) and ($I_{2},\;G_{2}$) tasks, the target object was barely picked up, and in the ($I_{3},\;G_{3}$) task, the target object was lifted close to the target height with much difficulty but wandered from the target trajectory, resulting in failure. The trajectories resulting from the policy learned through RL show that it could lift the target object to a certain height in the $\left(I_{1},\;G_{1}\right)\;\mathrm{and}\;\left(I_{3},\;G_{3}\right)$ pick-up tasks but failed the final objective in all tasks, presumably because RL could not learn within the given time sufficiently well to properly execute the tasks. These experimental results prove HIL’s ability to learn high-quality action policies within a short learning time.

## 5. Conclusions

This study proposes an HIL framework as an efficient method for learning robotic manipulation tasks. To ensure efficient and flexible learning of action policies, the proposed HIL framework combines the advantages of BC and SC in a mutually complementary manner. Meanwhile, it overcomes the limitations of BC-based learning of action policies being limited to those similar to the demo dataset and those of SC-based learning requiring more training time and computational effort, which neutralizes its advantages of higher flexibility and broader coverage over BC. The proposed HIL framework uses an adaptive loss mixing approach to adaptively balance the contributions of the BC and SC losses depending on the degree of policy convergence. Furthermore, the proposed HIL framework utilizes pretrained dynamics networks as a way to enhance SC efficiency and performs stochastic state recovery during SC implementation to ensure stable training of the policy network. Additionally, a series of experiments was conducted using a simulated Jaco robotic hand, which verified the high learning efficiency and policy flexibility of the HIL framework.

Currently, the proposed HIL framework has several limitations. First, it requires multiple sequences of (state, action) pairs as demo trajectories. However, there are many domains where it is possible to record the observed states during demonstration, but not the executed actions or controls. To deal with such state-only demo trajectories, the current HIL learning must be extended further. Second, as a hybrid imitation learning approach, the proposed HIL framework cannot learn the optimal policy due to some low-quality or wrong demo data. Third, the current HIL framework can only be implemented in a simulated robotic environment. Therefore, it is planned to develop a relevant sim-to-real learning scheme to enable the transfer of action policies learned with the HIL framework in simulated robotic environments to a real robot in the physical environment. Last, the current HIL framework does not provide a facility to use live demonstrations performed by humans. It will be interesting to extend the HIL framework to support live human demonstrations using a remote control such as a 3D mouse.

## Figures and Tables

**Table 1:** Variable descriptions.

Variable	Description
$s_{t}^{E}$	Demo state
$a_{t}^{E}$	Demo action
$s_{t}$	Task state
$a_{t}$	Task action
S	State space
A	Action space
P	Stochastic state transition probability
T	Assigned robotic task
$c_{initial}$	Initial state condition
$c_{goal}$	Goal state condition
$\pi_{\theta }$	Stochastic policy network
d	Demo task trajectory
D	Demo dataset
l	Length of each demo task trajectory
$L_{BC}$	Behavior cloning (BC) loss
$L_{SC}$	State cloning (SC) loss
$L_{mix}$	Mixed loss
$d_{conv}$	Degree of policy convergence
$f_{\phi }$	Dynamics network
$\alpha_{e}$	Loss mixing weight
ρ	State recovery probability

**Table 2:** Performance comparison among the methods using different loss mixing weights.

	Task	Pick-Up		Pick-and-Place		Stack	
Loss Mixing		Success Rate	Time (min)	Success Rate	Time (min)	Success Rate	Time (min)
$\alpha_{i}=0$		0.58	112	0.48	128	0.16	135
$\alpha_{i}=0.3$		0.75	293	0.52	422	0.46	414
$\alpha_{i}=0.6$		0.72	393	0.78	456	0.72	545
$\alpha_{i}=1.0$		0.64	460	0.76	625	0.66	724
$\alpha_{i}=d_{conv}$		0.75	119	0.84	156	0.78	181

**Table 3:** Performance comparison depending on the state recovery probability ρ.

	Task	Pick-Up	Pick-and-Place	Stack
Recovery				
ρ = 1.0		0.64	0.46	0.52
ρ = 0.6		0.75	0.84	0.78
ρ = 0.3		0.816	0.75	0.76
ρ = 0		−	−	−

## Data Availability

The datasets used and/or analyzed during the current study are available from the corresponding author upon reasonable request.
